# Magnesium: Biochemistry, Nutrition, Detection, and Social Impact of Diseases Linked to Its Deficiency

**DOI:** 10.3390/nu13041136

**Published:** 2021-03-30

**Authors:** Diana Fiorentini, Concettina Cappadone, Giovanna Farruggia, Cecilia Prata

**Affiliations:** Department of Pharmacy and Biotechnology, Alma Mater Studiorum—University of Bologna, 40126 Bologna, Italy; diana.fiorentini@unibo.it (D.F.); concettina.cappadone@unibo.it (C.C.); cecilia.prata@unibo.it (C.P.)

**Keywords:** magnesium, nutrition, hypomagnesemia, mg deficiency, mg detection

## Abstract

Magnesium plays an important role in many physiological functions. Habitually low intakes of magnesium and in general the deficiency of this micronutrient induce changes in biochemical pathways that can increase the risk of illness and, in particular, chronic degenerative diseases. The assessment of magnesium status is consequently of great importance, however, its evaluation is difficult. The measurement of serum magnesium concentration is the most commonly used and readily available method for assessing magnesium status, even if serum levels have no reliable correlation with total body magnesium levels or concentrations in specific tissues. Therefore, this review offers an overview of recent insights into magnesium from multiple perspectives. Starting from a biochemical point of view, it aims at highlighting the risk due to insufficient uptake (frequently due to the low content of magnesium in the modern western diet), at suggesting strategies to reach the recommended dietary reference values, and at focusing on the importance of detecting physiological or pathological levels of magnesium in various body districts, in order to counteract the social impact of diseases linked to magnesium deficiency.

## 1. Introduction

Magnesium is the fourth most abundant element in the human body (Ca²^+^ > K^+^ > Na^+^ > Mg²^+^) and the second most abundant cation within the body’s cells after potassium. The human body contains 760 mg of magnesium at birth and this quantity increases to 5 g at around 4–5 months [1,2]. The total Mg^2+^ body amount varies between 20 and 28 g [2,3]. More than 99% of the total body Mg^2+^ is located in the intracellular space, mainly stored in bone (50–65%), where, together with calcium and phosphorus, it participates in the constitution of the skeleton, but also muscle, soft tissues, and organs (34–39%), whereas less than 1–2% is present in blood and extracellular fluids [4,5]. Magnesium concentration within erythrocytes is three times higher than in plasma [6], where normal magnesium concentrations range between 0.75 and 0.95 millimoles (mmol)/L [7]. Up to 70% of all plasma Mg^2+^ exists in the ionized (free) active form [8]. A serum magnesium level less than 1.7–1.8 mg/dL (0.75 mmol/L) is a condition defined as hypomagnesemia [9]. Magnesium levels superior to 2.07 mg/dL (0.85 mmol/L) are most likely linked to systemic adequate magnesium levels [10,11], as also reported by Razzaque, who in addition suggests to individuals with serum magnesium levels between 0.75 to 0.85 mmol/L to undergo further investigation to confirm body magnesium status [12]. In addition, a urinary excretion < 80 mg/*die* could indicate a risk of magnesium deficiency, because in this condition renal excretion decreases as a compensatory mechanism [13].

Analogously to calcium, body magnesium content is physiologically regulated through three main mechanisms: intestinal absorption, renal re-absorption/excretion, and exchange from the body pool of magnesium (i.e., bones). Mg^2+^ stores are indeed tightly regulated via a balanced interplay between intestinal absorption and renal excretion under normal conditions. The elimination of magnesium by the kidneys increases of course when there is a magnesium surplus and can decreases to just 1 mEq of magnesium (~12 mg) in the urine during deficits. Despite renal conservation, magnesium can be derived from the bone (as well as muscles and internal organs) in order to preserve normal serum magnesium levels when intakes are low, as with calcium [14]. Mg^2+^ insufficient levels have been documented in ill patients since the end of the last century [15]. Nevertheless, despite the well-recognized importance of magnesium, Mg^2+^ availability is not generally determined and monitored in patients, therefore, magnesium has been called the “forgotten cation” [16,17]. Moreover, serum magnesium levels do not usually reflect the content of magnesium in different body districts. Therefore, a normal level of serum magnesium does not rule out magnesium deficiency [18]. In the last 20–30 years, a great number of epidemiological, clinical, and experimental research papers have shown that abnormalities of magnesium levels, such as hypomagnesemia and/or chronic magnesium deficiency, can result in disturbances in nearly every organ/body, contributing to or exacerbating pathological consequences and causing potentially fatal complications [19,20,21].

Magnesium subclinical deficiency is not uncommon among the general population [18]. Although kidneys limit urinary excretion of this mineral to avoid hypomagnesemia, habitual low intakes of magnesium or excessive losses, due to different causes and conditions, can lead to a magnesium subclinical deficiency. Early signs of magnesium deficiency include weakness, loss of appetite, fatigue, nausea, and vomiting. Afterwards, muscle contractions and cramps, numbness, tingling, personality changes, coronary spasms, abnormal heart rhythms, and seizures can occur when magnesium deficiency worsens [22]. Finally, severe magnesium deficiency can result in hypocalcemia or hypokalemia because mineral homeostasis is disrupted [23].

As previously cited, hypomagnesaemia is usually defined as serum magnesium concentration <0.75 mmol/L [21], nevertheless, there are various concerns about the use of this parameter as a marker of real magnesium content in cells/body [9].

This review focuses on the biochemical mechanisms underneath magnesium cellular functions, in order to elucidate the correlation with the potential risks associated to magnesium deficiency. Moreover, other important topics to be addressed are the nutritional strategies that enable the prevention of low magnesium intake and the methods to detect when magnesium content is truly physiological or pathological. Therefore, a comprehensive analysis of the data available on magnesium content and bioavailability (i.e., the fraction of an ingested nutrient that becomes available for use and storage in the body) is here reported together with a discussion about the available methods for the evaluation of magnesium content in the human organism.

Finally, a survey on the social impact of the principal diseases linked to magnesium deficiency is reported, focusing in particular on diabetes, osteoporosis, cardiovascular, and neurological diseases and cancer.

## 2. Biochemistry of Magnesium to Understand the Consequences of Its Deficiencies

Magnesium (atomic number 12, atomic mass 24.30 Da) is classed as an alkaline earth metal belonging to the second group of the periodic table of the elements. Like calcium, its oxidation state is 2+ and, owing to its strong reactivity, it frequently occurs as free cation Mg^2+^ in aqueous solution or as the mineral part of a substantial variety of compounds, including chlorides, carbonates, and hydroxides rather than in a native metallic state.

Mg^2+^ is mainly absorbed through the small intestine, although some is also taken up through the large intestine. There are two known transport systems for Mg^2+^, a passive paracellular mechanism and transcellular transport by dedicated Mg^2+^ channels and transporters. In particular, member 1 of solute carrier family 41 (SLC41A1), magnesium transporter 1 (MagT1), and transient receptor potential melastatin type 6 and 7 (TRPM6 and TRPM7) have been described. The role of these Mg^2+^ transporters in the establishment of Mg^2+^ homeostasis and the molecular mechanism of their action have been reviewed in detail in several recent papers present in the literature [2,24,25,26,27,28,29,30,31,32]. Mg^2+^ homeostasis is maintained by the intestine, bone, and kidneys under hormonal control. Briefly, serum Mg^2+^ is filtered by the renal glomeruli and then reabsorbed along the nephron, where the reabsorption pathways differ in each segment. Magnesium transport across cell membranes shows tissue variability and, among the body’s tissues, is higher in the heart, liver, kidney, skeletal muscle, red cells, and brain. Thus, magnesium transport, the physiology of magnesium homeostasis, and metabolic activity of the cell are strictly correlated [19,23,25,33,34,35].

The level of renal excretion of Mg^2+^ mainly depends on the serum Mg^2+^ concentration. Blood magnesium concentrations are strictly regulated in order to maintain a normal range even if the dietary magnesium intakes are low or an excessive magnesium excretion occurs. While serum/plasma magnesium concentrations remain in the healthy range however, both bone and soft tissue intracellular magnesium concentrations may be depleted [7]. Unlike other ions, for which cells are observed to maintain transmembrane gradients, cellular and extracellular concentrations of free Mg^2+^ are similar. Mg^2+^ typical intracellular concentrations range from 10 to 30 mM. However, since most Mg^2+^ is automatically associated with ribosomes, polynucleotides, ATP, and proteins upon its entry into the cells, its freely available concentration falls varies between 0.5 and 1.2 mM [36]. 

Approximately half of magnesium present in the body can be found in bone, 30% of which is exchangeable and functions as a pool to stabilize serum Mg^2+^ concentration [37]. Mg^2+^ is an integral part of apatite crystals, from which it is released in the course of bone resorption. The other half of magnesium is localized in soft tissue, with <1% present in the blood. 

Mg^2+^ is involved in practically every major metabolic and biochemical process within the cell and is responsible for numerous functions in the body, including bone development, neuromuscular function, signaling pathways, energy storage and transfer, glucose, lipid and protein metabolism, DNA and RNA stability, and cell proliferation. 

Over 600 enzymes with Mg^2+^ as a cofactor are currently listed by the enzymatic databases, while an additional 200 are listed in which Mg^2+^ may act as an activator [38,39]. More specifically, it mainly interacts directly with the substrate, rather than acting as a real cofactor.

**Figure 1:**
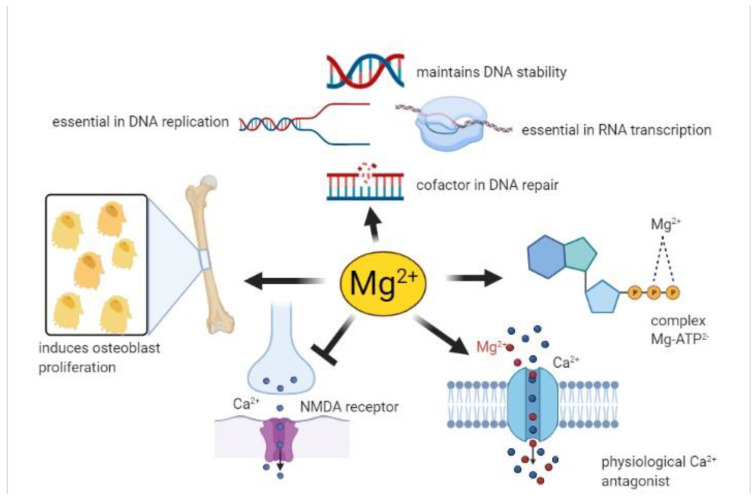
The biochemical involvement of magnesium in many cellular processes. This image is created with BioRender.com.

The involvement of magnesium in many cellular processes (Figure 1) is here briefly summarized and then detailed in the following paragraphs, explaining why habitually low intakes of magnesium induce changes in biochemical pathways that can lead to an increased risk of illness over time.

− The complex MgATP^2-^ is required for the activity of many enzymes. In general, Mg^2+^ acts as a cofactor in all reactions involving the utilization and transfer of ATP, including cellular responses to growth factors and cell proliferation, being thus implicated in virtually every process in the cells. Mg^2+^ availability is a critical issue for carbohydrate metabolism, which may explain its role in diabetes mellitus type 2 [40];
− Mg^2+^ is necessary for the correct structure and activity of DNA and RNA polymerases [41,42]. In addition, topoisomerases, helicases, exonucleases, and large groups of ATPases require Mg^2+^ for their activity, therefore Mg^2+^ is essential in DNA replication, RNA transcription, and protein formation, being thus involved in the control of cell proliferation. Moreover, Mg^2+^ is crucial to maintain genomic and genetic stability, stabilizing the natural DNA conformation and acting as a cofactor for almost every enzyme involved in nucleotide and base excision repair and mismatch repair. Given these effects, low Mg^2+^ availability can be involved in the development of cancer [2];
− Serum Mg^2+^ concentrations are strongly related to bone metabolism; bone surface Mg^2+^ is constantly exchanged with blood Mg^2+^ [43,44]. Furthermore, Mg^2+^ induces osteoblast proliferation [45] therefore, the consequences of Mg^2+^ deficiency are accelerated bone loss and a decline in bone formation [46];
− Mg^2+^ participates in controlling the activity of some ionic channels in many tissues. Its mechanism of action relies on either direct interaction with the channel, or an indirect modification of channel function through other proteins (e.g., enzymes or G proteins), or via membrane surface charges and phospholipids [47]. Furthermore, Mg^2+^ acts as a physiological Ca^2+^ antagonist within cells, since it can compete with Ca^2+^ for binding sites in proteins and Ca^2+^ transporters [48]. These abilities are involved in the observed effect of magnesium on the cardiovascular system, muscle, and brain;
− Neuronal magnesium concentrations downregulate the excitability of the N-methyl-D-aspartate (NMDA) receptor, which is essential for excitatory synaptic transmission and neuronal plasticity in learning and memory [49]. Magnesium blocks the calcium channel in the NMDA receptor and must be removed for glutamatergic excitatory signaling. Low serum Mg^2+^ levels increase NMDA receptor activity thus enhancing Ca^2+^ and Na^+^ influx and neuronal excitability. For these reasons, a deficiency of Mg^2+^ has been hypothesized in many neurological disorders, such as migraine, chronic pain, epilepsy, Alzheimer’s, Parkinson’s, and stroke, as well as anxiety and depression [50].

### 2.1. Magnesium as Enzymatic Cofactor

As stated above, magnesium is a cofactor for over 600 enzymes and an activator for an additional 200 enzymes [51]. Given the ability of Mg^2+^ to bind inorganic phosphate, ATP, phosphocreatine, and other phosphometabolites make complex with magnesium, with important consequences for many metabolic reactions, especially those related to carbohydrate metabolism and cellular bioenergetics. The binding between ATP and Mg^2+^ results in an adequate conformation that allow to weaken the terminal O–P bond of ATP, thereby facilitating the transfer of phosphate [14,52].

The paramount importance of magnesium in the glycolytic pathway [53] and mitochondrial synthesis of ATP [54] has been known for a long time. Many of the glycolytic enzymes are sensitive to magnesium, whose principal function is to facilitate the transfer of high energy phosphate. Thus, hexokinase, phosphofructokinase, phosphoglycerate kinase, and pyruvate kinase work in this manner, while aldolase and enolase require Mg^2+^ for their stability and activity [53].

In mitochondria, the activity of three important dehydrogenases is dependent on Mg^2+^. Isocitrate dehydrogenase is directly stimulated by the Mg^2+^-isocitrate complex [55], α-ketoglutarate dehydrogenase complex by free Mg^2+^ [56], pyruvate dehydrogenase is indirectly stimulated via the stimulatory effect of Mg^2+^ on pyruvate dehydrogenase phosphatase, which dephosphorylates and thus activates the pyruvate dehydrogenase complex [57]. Furthermore, Mg^2+^ has been shown to be the activator of ATP synthesis by mitochondrial F_o_/F_1_-ATPase [58]. Furthermore, it has been demonstrated that Mg^2+^ concentration is low in the brain of patients affected by mitochondrial cytopathies and that supplementation with Coenzyme Q_10_ has improved oxidative phosphorylation and the cytosolic magnesium level [59].

The enzyme creatine kinase, catalyzing the reversible reaction between creatine phosphate and ADP to form creatine and ATP, is strongly influenced by the concentration of free Mg^2+^. This enzyme can synthesize ATP when the muscle or heart are subjected to a heavy workload and can be localized both in cytosol and mitochondrion [53].

In the liver, Mg^2+^ is an important regulator of gluconeogenic enzymes, including glucose-6-phosphatase and phosphoenolpyruvate carboxykinase [40].

The fundamental role of Mg^2+^ in glycolysis/gluconeogenesis, pentose phosphate pathway, and Krebs cycle was also demonstrated in a metabolomic analysis of the liver of rats fed a magnesium-deficient diet. Results of this study show that hepatic contents of glucose-6-phosphate, citric, fumaric, and malic acids were significantly reduced in animals fed a magnesium-deficient diet, and also fructose-6-phosphate and succinic acid were numerically lower. By contrast, the mRNA levels of the related enzymes, such as glucokinase, glucose-6-phosphatase, glucose-6-phosphate dehydrogenase, and phosphoenolpyruvate carboxykinase were not significantly different between control and treated animals. These data demonstrated that, in the liver, the metabolite content associated to glucose metabolism was altered by a magnesium deficiency [60], therefore a balanced magnesium status seems an important requisite for adequate carbohydrate metabolism [61].

In this regard, magnesium deficiency has been correlated to type 2 diabetes mellitus, metabolic syndrome, and insulin resistance [62]. As well as all other protein kinases, the tyrosine kinase activity of the β-subunit of the insulin receptor is dependent on magnesium concentration, therefore a magnesium deficiency may result in an impaired insulin signal. In other words, the lower the basal magnesium, the greater the amount of insulin required to metabolize the same glucose load, indicating decreased insulin sensitivity [62,63]. A recent study demonstrates that magnesium improves glucose consumption and glucose tolerance through two main mechanisms: stimulation of the GLUT4 gene expression and translocation of this glucose transporter to the plasma membrane, and suppression of the glucagon effect and gluconeogenesis pathway in the liver and muscle [64]. Table 1 summarizes the principal enzymes involved in carbohydrate metabolism which require magnesium for their proper action.

### 2.2. Magnesium and Nucleic Acids

Divalent cations are widely known to affect the structure of duplex DNA. The possibility of hydrogen bond interactions between cations and DNA is greater for divalent ions, owing to their hydration properties. Mg^2+^ ion attracts negatively-charged DNA phosphate groups and with its six-coordinated water molecules forms hydrogen bonds, locally reducing the DNA negative charge density and stabilizing its structure and natural conformation [66]. This magnesium effect may be named the ‘protective effect’. However, when magnesium binds ‘covalently’ to DNA, it forms a bound coordination complex, causing a local distortion of the double helix, which may lead to the destruction of the cell. This effect occurs at higher concentrations of magnesium, therefore, the maintenance of the cellular Mg^2+^ concentration within the physiological range is essential for DNA stability [66].

As stated above, Mg^2+^ also has an important role in DNA repair mechanisms. Several enzymes of these systems depend on magnesium, some of them are common to different repair systems, such as DNA polymerase beta, DNA ligases, and DNA endonucleases, while others are specific to one repairing mechanism [67].

All DNA polymerases catalyze the same nucleotidyl-transfer reaction, forming and breaking phosphodiester bonds, and all DNA polymerases contain two-three conserved carboxylates in the catalytic center. The Mg^2+^ ions neutralize the charge of the catalytic carboxylates and triphosphates of dNTP, thus facilitating the alignment of the substrates for the chemical reaction [68]. Moreover, other important nuclear enzyme activities involved in DNA replication depend on magnesium, like topoisomerases, helicases, and exonucleases. Thus, it may be concluded that magnesium is essential for DNA structure, duplication and repair, as well as maintaining genomic and genetic stability. Hence, magnesium deficiency could favor DNA mutations leading to the initiation of carcinogenesis [67]. 

As far as RNA is concerned, the role of Mg^2+^ in restoring denatured tRNA molecules was reported in 1966 [69]. Later, the importance of Mg^2+^ binding for the tRNA tertiary structure has been debated over the years, questioning the prevalent action of nonspecific diffuse binding of Mg^2+^ (or other divalent cations) or a specific Mg^2+^ interaction [2,70,71]. A very recent paper demonstrated that magnesium ions are required by tRNAPhe for proper recognition of UUC/UUU codons during ribosomal interactions with tRNA [72]. 

It has been recently demonstrated that the biological environment and biomolecules can stimulate RNA functions [73]. To this regard, amino acids and nucleotides are abundant cellular metabolites, and it has been shown that cellular concentrations of amino acid-chelated Mg^2+^ stimulate RNA folding and catalysis [74]. The authors hypothesized that the amino acid-bound Mg^2+^ ion may interact with the RNA, “sharing” the Mg^2+^ ion with it, thus decreasing the folding free energy of RNA, stabilizing the RNA structure, and promoting RNA high catalytic activity [74]. Furthermore, the same authors demonstrated that also nucleotide diphosphate-chelated Mg^2+^ promotes RNA catalysis much like amino acid-bound Mg^2+^ [75]. The authors observed that the stimulatory effects of Mg^2+^ diphosphate-containing metabolites is general for RNA and DNA enzymes [75].

Moreover, RNA synthesis require two Mg^2+^ ions for the catalysis [68], thus magnesium is also involved in RNA transcription. The aspartate triad, three aspartate residues which reside at the center of the active site, is absolutely conserved among all DNA-dependent multi-subunit RNA polymerase. These amino acids chelate the first of two essential magnesium ions required for catalysis, the second ion is brought into the active center bound to the incoming nucleotide substrate [76].

Traduction is also highly dependent on intracellular magnesium concentration, it has been suggested that, upon growth factor binding to their receptors, Mg^2+^ enters the cell and the increased cytosolic Mg^2+^ level contributes to ribosomal activity and protein synthesis. Moreover, it should not be forgotten that the activities of receptor and non-receptors tyrosine kinases have an obligatory requirement for Mg^2+^, thus involving magnesium in the signaling pathways of growth factors, such as VEGF, EGF, PDGF, and so on. Therefore, Mg^2+^ is an important factor in controlling cell proliferation [77].

Given these considerations, it is reasonable to hypothesize the involvement of magnesium in tumor growth, owing to the ability of Mg^2+^ to regulate several cancer-associated enzymes, particularly those involved in glycolysis—the preferred pathway used by neoplastic cells to produce energy—and DNA repair. On the other hand, low dietary Mg^2+^ intake has been associated with the risk of several types of cancers, extensively reviewed by Castiglioni [78].

### 2.3. Magnesium and Bone Metabolism 

It has been observed that bones belonging to magnesium-deficient animals are brittle and fragile, microfractures of the trabeculae can be detected and mechanical properties are severely impaired [79]. In general, all experimental data obtained from animal studies indicate that the reduced dietary intake of magnesium is a risk factor for osteoporosis through a variety of different mechanisms [80]. Mg^2+^ increases the solubility of the minerals, which constitute the hydroxyapatite crystals, such as Pi and Ca^2+^, thereby acting on crystal size and formation [81]. It has been demonstrated that osteoporotic women with a magnesium deficiency have larger and better organized crystals in trabecular bone than controls, making bone more susceptible to fractures [82].

Besides its direct effect on hydroxyapatite crystals, other mechanisms are involved in the structural role of magnesium and bone health. Of great importance is the complex interplay existing between magnesium and vitamin D: vitamin D stimulates intestinal magnesium absorption [83] and magnesium deficiency reduces the levels of 1,25(OH)_2_D_3_, thus being implicated in magnesium-dependent vitamin D-resistant rickets [84]. In fact, magnesium is required for the activity of hepatic 25-hydroxylase and renal 1α-hydroxylase [85], both crucial to convert 25(OH)D into its biologically active form 1,25(OH)_2_D_3_, and also to facilitate the transfer of vitamin D to target tissues through the vitamin D binding protein [86]. On the other hand, magnesium is also involved in the inactivation of vitamin D, being required for the activity of the renal 24-hydroxylase, to form 24,25(OH)_2_D [87]. 

Another complex relationship occurs between magnesium and parathyroid hormone (PTH), and thus indirectly between magnesium and calcium. PTH is secreted by parathyroid glands in response to a low level of serum calcium and either high or low PTH levels can result in calcium dysregulation and bone disease. Particularly, a chronic sustained activation of the receptor by PTH, as observed in primary hyperparathyroidism, exerts catabolic effects on bone and leads to an enhanced bone turnover, resulting in bone loss and an increased fracture risk [88]. Physiological serum calcium level negatively regulates PTH secretion, but also the serum level of PTH and magnesium are co-dependent. Low levels of magnesium stimulate the secretion of PTH, but very low magnesium concentration inhibits PTH secretion [89]. Magnesium can also reduce PTH secretion at low calcium concentrations [90]. Interestingly, magnesium is also required for the sensitivity of the target organs to PTH signal [21] and impaired peripheral response to PTH leads to a low serum concentration of vitamin D [91]. 

Additionally, it has been demonstrated that magnesium deficiency in animal models induces a clinically inflammatory syndrome, characterized by leukocyte and macrophage activation, the release of inflammatory cytokines, and excessive production of free radicals. Since magnesium acts as a natural calcium antagonist, the molecular basis for inflammatory response could be the result of modulation of intracellular calcium concentration [92]. Many studies have demonstrated that in humans, a moderate or subclinical magnesium deficiency can induce chronic low-grade inflammation or exacerbate inflammatory stress caused by other factors [93]. This low-grade inflammation increases the secretion of pro-inflammatory cytokines, which stimulate the resorption of bone by the induction of the differentiation of osteoclasts from their precursors [94]. The ability of Mg^2+^ to decrease the inflammatory response and oxidative stress, as well as improving lung inflammation, possibly by inhibiting IL-6 pathway, NF-κB pathway, and L-type calcium channels [95], has raised the hypothesis of a possible magnesium supplementation in the prevention and treatment of COVID-19 patients, as suggested in the recent papers by Tang [96] and Iotti [97].

### 2.4. Magnesium, Calcium, and Cardiovascular System

As previously mentioned, intricate interactions between magnesium and calcium exist, and it has long been known that calcium intake affects magnesium retention and vice versa. As stated above, the Mg^2+^/Ca^2+^ ratio is very important for the activity of Ca^2+^-ATPases and other Ca^2+^ transporting proteins [48], thus small changes in the Mg^2+^ availability within the cell may cause perturbed Ca^2+^ signaling.

Magnesium plays an important role in the cardiovascular system, influencing myocardial metabolism, Ca^2+^ homeostasis, and endothelium-dependent vasodilation. It also acts as an antihypertensive, antidysrhythmic, anti-inflammatory, and anticoagulant agent. In myocardium, the opening of L-type Ca^2+^ channels produces a long-lasting Ca^2+^ current, corresponding to the second phase of the cardiac action potential. Mg^2+^ inhibits these channels, preventing Ca^2+^ overload and cell toxicity and thus exerting a myocardial protective effect [98]. Two general mechanisms could explain how Mg^2+^ regulates Ca^2+^ fluxes through L-type channels: alteration of ion permeation and/or modulation of channel gating properties. L-type Ca^2+^ channel gating is, in turn, regulated by membrane potential, cytosolic Ca^2+^ concentration, and channel phosphorylation. It has been demonstrated that the effect of Mg^2+^ is dependent on the channel’s phosphorylation state, since phosphatase treatment decreases the inhibitory effect of Mg^2+^ [99]. Furthermore, Mg^2+^ is necessary for Na^+^/K^+^-ATPase, which is responsible for the active transport of K^+^ intracellularly during the action potential duration. Mg^2+^ is also involved in regulating the K^+^ influx through different K^+^ channels [100]. The modulation of cardiac action potential can explain the antidysrhythmic action of Mg^2+^: Its infusion provokes the slowing of atrioventricular nodal conduction, and also determines the prolongation of PR interval and QRS duration in the electrocardiogram [101]. The effect of Mg^2+^ on cardiomyocytes also depends on other mechanisms, including the ability of Mg^2+^ to compete with Ca^2+^ for binding sites in proteins, such as calmodulin, troponin C, and parvalbumin [48], to act as substrate in a complex with ATP for cardiac Ca^2+^-ATPases, and to alter the affinity of Na^+^-Ca^2+^ exchanger [2]. In summary, tight regulation of Mg^2+^ concentration in myocytes is necessary for optimal cardiac function, indeed hypomagnesemia can impact physiological activity, leading to cardiovascular diseases [102].

A relationship between Mg^2+^ levels and blood pressure has been established [2,21,103]. Many different mechanisms are involved in the Mg^2+^ vasodilation effect, among which the ability of Mg^2+^ to act as a natural calcium channel blocker [104] and to upregulate the endothelial nitric oxide synthase, thus increasing nitric oxide (NO) release [102]. Additionally, Mg^2+^ is able to increase the production of prostacyclin (PGI_2_), a platelet inhibiting factor [2], it is involved in a step of the synthesis of prostaglandin E_1_ (PGE_1_), a vasodilator and platelet inhibitor agent [104], and owing to its anti-inflammatory role, Mg^2+^ results in an improved lipid profile, reduced free oxygen radicals, and improved endothelial function [2]. 

Finally, the antagonistic action of Mg^2+^ on calcium channels and Ca^2+^-binding proteins has been related to muscle cramps and spasms observed as recurrent symptoms in patients with hypomagnesia [105]. In this regard, conflicting results have been published about magnesium supplementation in clinically cramp prophylaxis [106]. 

### 2.5. Magnesium, Calcium, and Brain

One of the main neurological functions of magnesium is due to magnesium’s interaction with the N-methyl-D-aspartate (NMDA) receptor. NMDA receptors are activated upon glutamate binding and mediate the influx of Ca^2+^ and Na^+^ ions and the efflux of K^+^ ions. Glutamate is the major excitatory neurotransmitter in the brain, acting by binding to various transporters and receptors, among which are the cation channels AMPA (2-amino-3-(3-hydroxy-5-methyl-isoxazol-4-yl) propanoic acid) and NMDA. The NMDA receptor family includes a wide variety of receptor subtypes, both diheteromers and triheteromers, each with distinct biophysical, pharmacological, and signaling properties and different locations between brain regions [107]. Going into the detail of Mg^2+^ mechanism of action, glutamate from presynaptic neuron binds both AMPA and NMDA receptors on the postsynaptic neuron, but at a normal membrane potential, Mg^2+^ ions block NMDA receptors, allowing only AMPA receptor activation to occur. When the membrane potential rises, NMDA receptors are unlocked, and they facilitate the cation influx upon glutamate binding. When Mg^2+^ concentration is reduced, less NMDA channels are blocked and this increased excitatory postsynaptic potential causes hyperexcitability of the neurons, which can lead to oxidative stress and neuronal cell death [2]. Another mechanism involving Mg^2+^ in neuron hyperexcitability is its ability to regulate the function of the inhibitory GABA receptors [108]. In case of low Mg^2+^ concentration, the membrane potential will be higher, thus relieving Mg^2+^ block of the NMDA receptors and contributing to hyperexcitability of the neurons. 

Noteworthy, abnormal glutamatergic neurotransmission has been implicated in many neurological and psychiatric disorders, including migraine, chronic pain, epilepsy, Alzheimer’s, Parkinson’s, depression, and anxiety [109].

Furthermore, a NMDA receptor-dependent mechanism is involved in the Mg^2+^ dependent enhancement of the activity of nitric oxide synthases, causing the NO release, which has multiple functions in the brain, such as vasodilation, regulation of gene transcription, and neurotransmitter release [110]. Mg^2+^ also increases the expression and secretion of calcitonin gene-related peptide (CGRP), which has a vasodilatory effect [111], while Mg^2+^ deficiency increases the release of substance P, a neuroinflammatory agent, which stimulates the secretion of inflammatory mediators [112]. Hence, Mg^2+^ has also a role in the regulation of neuropeptides release and oxidative stress, contributing to the maintenance of a healthy neurological function. 

## 3. Nutritional Strategies to Avoid Magnesium Deficiencies 

Magnesium is an essential nutrient for living organisms therefore it must be supplied regularly from our diet to reach the recommended intake, preventing deficiency. Consequently, it is important not only to identify the possible sources of magnesium, but also to assess the bioavailability and factors that can influence its absorption and elimination.

### 3.1. Recommended Intake and Categories of People That Risk Inadequate Magnesium Intake

Intake recommendations for magnesium and other nutrients have been provided by the World Health Organization (WHO) and the Food and Agriculture Organization (FAO), by the American National Academy of Medicine (NAM), previously called the Institute of Medicine (IoM), and by the European Food Safety Agency (EFSA). According to the development of scientific knowledge about the roles played by nutrients in human health, the Food and Nutrition Board at the National Academy, with the Health Canada partnership, has updated what used to be known as Recommended Dietary Allowances (RDAs) and renamed a new version of these guidelines as “Dietary Reference Intakes” (DRIs) [3,113,114]. Analogously, the European Food Safety Agency (EFSA) provides Dietary Reference Values (DRVs) [115,116]. LARN (“Livelli di Assunzione di Riferimento di Nutrienti ed energia per la popolazione italiana” corresponding to “Recommended Levels of Nutrients and Energy Intakes”) are the last version of the Italian DRVs, more recently released in 2014 by the Italian Society of Human Nutrition and periodically updated by the Commission of the Human Nutrition Society (SINU) and by the Ministry of Agricultural, Food, and Forestry Policies (CREA), in line with the EFSA technical reports [16,117,118,119].

These values, which vary according to sex and specific-age ranges, can be used to identify nutrient intakes that are relevant for diet planning in individuals as well as in the general population and include:

− The population reference intakes (PRIs), which refer to the level of nutrient intake that is adequate for the majority of people in a population group;
− The average requirements (ARs), which refer to the intake level that is adequate to meet the physiological requirements of 50% of healthy individuals. This parameter is usually taken into consideration not only to assess the nutrient intakes of groups of people and to plan nutritionally adequate diets for them but also to assess the nutrient intakes of individuals.

In case of insufficient scientific evidence to estimate the AR and/or PRI, adequate intake (AI) is established by estimating the intake of an apparently healthy population group that is assumed to have adequate intake.

− Adequate intake (AI), therefore, refers to the intake assumed to ensure nutritional adequacy;
− Tolerable upper intake level (UL): Maximum daily intake which is considered to be safe/without adverse health effects on the totality of the considered population.

The current intake recommendations for magnesium are reported in Table 2.

Magnesium adequate intake for infants from birth to 12 months is determined by considering the mean intake of magnesium in healthy, breastfed infants, with added solid foods during the first 7–12 months of life.

The gradual transition from an exclusively milk-based diet to one including a different range of family foods that occurs during the 6–24 months of life, requires a consumption of a healthy and balanced diet. Although an adequate intake of micronutrients is clearly critical during this sensitive period of growth and development [120], insufficient intake of some micronutrients are observed also in industrialized countries. Regarding magnesium, recommendations on infant requirements from WHO/FAO, American National Academy of Medicine, and EFSA were based on estimations of intake [116,121,122]. There is insufficient information on either magnesium or phosphorus to establish a UL for infants and young children (0–3 years old).

Considering all the evidence available from prospective observational studies and balance studies, the EFSA Panel, in the last version of Scientific Opinion on Dietary Reference Values (DRV) for magnesium (2015) [116], decided to set an AI based on observed intakes in nine European Union countries (Italy, Finland, France, Germany, Ireland, Latvia, the Netherlands, Sweden, and the UK). The panel proposed to set AIs according to sex, for adults of all ages. Considering the distribution of observed average intakes, the panel proposed AI values according to sex and ages, as reported in Table 2. 

There are several international guidelines that give advice for the general population in order to maintain a healthy status therefore, when specifying or describing an advisory amount of macro-micronutrients it is important to indicate what system and/or updated sources has been used because the values are similar but not always the same. For example, magnesium requirements (RDA) for adults (18–29 years) in Japan are 340–370 and 270–290 mg/die for male and female, respectively [115,123].

During pregnancy and lactation, the correct uptake of magnesium is particularly important as evidenced by Durlach J. [124]. Nevertheless, since pregnancy causes only a small increase in magnesium requirement, which is probably satisfied by adaptive physiological mechanisms, the EFSA panel considers the same AI for nonpregnant and pregnant women. Data from the Cochrane Database of Systematic Reviews suggest that there is not enough high-quality evidence to show that dietary magnesium supplementation during pregnancy is beneficial, as deeply analyzed and reported by Makrides and Colleagues [125].

Analogously, taking into consideration that 25 mg/day is secreted with breast milk during the first six months of exclusive breastfeeding and that there is the strategy adaptation of magnesium metabolism, at the level of both absorption and elimination, the panel considers the same AI both for non-lactating and lactating women. The Italian LARN are in agreement with these considerations, although the values are lower than those suggested by the EFSA.

Athletes are recommended to consume higher amounts of potassium and magnesium. In particular, 420 mg/die for male and 320 mg/die for female, considering 19–50 years as an age range [18]. Usually, renal elimination involves approximately 100 mg of Mg^2+^ per die, whereas the losses via sweat are generally low. However, during intense exercise, these losses can increase considerably. Moreover, since Mg^2+^ activates the enzymes involved in protein synthesis, it is involved in ATP metabolism and Mg^2+^ serum levels decrease with exercise, magnesium supplementation could therefore improve energy metabolism and ATP availability. Magnesium supplementation generally does not affect an athlete’s performance, unless there is a deficiency state [126,127,128,129,130], although this result seems to be disproved by a recent double-blind study carried out by Reno A.M. and colleagues [131]. This double-blind study, although with several criticisms (e.g., small number of subjects and absence of initial magnesium status detection), shows that magnesium supplementation (vs. Pla) significantly reduced muscle soreness and improved perceived recovery. As far as the role of magnesium against skeletal muscle cramps is concerned, a recent update of a Cochrane Review that assessed evaluating magnesium for exercise-associated muscle cramps or disease-state-associated muscle cramps was found. Moreover, magnesium supplementation did not provide clinically meaningful cramp prophylaxis to older adults experiencing skeletal muscle cramps. Further research is also needed to also evaluate the protective effect of magnesium [106].

Besides athletes, the following groups of people suffer more frequently from magnesium deficiency:

− Older people absorb less magnesium from the gut and lose more magnesium because of an increased renal excretion. Chronic magnesium deficiency is indeed common in the elderly, usually due to a decrease both in diet assumption and intestinal absorption, and it is probably exacerbated by estrogen deficit, which occurs in aging women and men and cause hypermagnesuria [132]. In a very recent and comprehensive review [34], Lo Piano and colleagues highlight the risk and consequences of the reduce intake and absorption of magnesium by elderly people;
− People affected by gastrointestinal diseases with consequent general malabsorption, such as Crohn’s disease [117,133,134,135,136,137,138,139,140], inflammatory bowel diseases [135,138,140,141], and celiac disease [142,143,144,145,146,147,148,149,150]. In particular, besides the absorption inefficiency due to celiac disease, a gluten free-diet was found to be poor in fiber and micronutrients, such as magnesium [151,152]. Therefore, people suffering from celiac disease are a typical example of subjects particularly susceptible to magnesium deficiency as they are simultaneously exposed to two risk factors;
− People affected by type 2 diabetes, although it still remains unclear if magnesium deficiency represents a cause or a consequences of this pathology [3,21,135,153,154,155,156];
− People who used to drink alcohol/alcoholics or are affected by long-term alcoholism [3,157,158,159,160] and are therefore affected by intestinal malabsorption. Spirits (such as brandy, cognac, gin, rum, vodka, and whisky) do not contain significant traces of magnesium. Moderate alcohol consumption, such as wine and beer during meals, is acceptable and is also included in the Mediterranean food pyramid (2–4 units/day), however, despite beer and wine having magnesium levels that range from 30–250 mg/L and fermented apple ciders ranging from 10–50 mg/L, such beverages cannot be considered as a reliable source of magnesium because they cause magnesiuresis and can have a laxative effect, with consequent problems on bioavailability and absorption. Ethanol is indeed magnesiuretic by causing proximal tubular dysfunction and increasing urinary magnesium loss, and its effect is rapid and common in people with an already negative magnesium balance [6,161];
− People under treatment with drugs (e.g., diuretics, proton pump inhibitors, tacrolimus, an immunosuppressor, chemotherapeutic agents, and some phosphate-based drugs) [6].

However, it is important to point out that most apparently healthy people risk an insufficient magnesium intake due to a decreased presence of this metal in the modern Western diet characterized by a wide use of demineralized water, processed foods, and agricultural practices that use soil deficient in magnesium for growing food [162,163,164], as discussed in the next paragraph and reported for the Spanish population, where about 75% of the population revealed intakes below 80% of the national and European recommended daily intakes [165]. Accordingly, data on people’s dietary habits still reveal that intakes of magnesium are lower than the recommended amounts either in the United States or in Europe. Epidemiological studies have shown that people consuming Western-type diets introduce an insufficient amount of micronutrients and in particular, a quantity of magnesium that is <30–50% of the RDA. Accordingly, the magnesium dietary intakes in the United States have been decreasing over the last 100 years from about 500 mg/day to 175–225 mg/day [21], and a general similar decrease in magnesium daily uptake in people fed a Western diet is reported in a recent and interesting review by Cazzola and Colleagues [164].

### 3.2. Magnesium Food Content and Bioavailability

Magnesium is considered widely distributed in foods, although the amount of magnesium contained in food is influenced by various factors including the soil and water used to irrigate, fertilizers, conservation, and also refining, processing, and cooking methods. In general, seeds, legumes, nuts (almonds, cashews, Brazil nuts, and peanuts), whole grain breads, and cereals (brown rice, millet), some fruits, and cocoa are considered good sources of magnesium. Nevertheless, acidic, light, and sandy soil is usually deficient in magnesium content. Moreover, agricultural techniques, such the use of potassium and ammonium at high concentration in fertilizers lead to magnesium depletion in food [1] and a recent meta-analysis on the effects of magnesium fertilization has been recently published [166]. 

Green leafy vegetables are frequently counted among the food rich in magnesium according to the hypothesis that chlorophyll-bound magnesium may represent important nutritional sources of magnesium. This hypothesis relies on what is known about iron, which is similarly bound in the porphyrin ring of heme, and is absorbed to a greater extent than non-heme iron. This concept is incorrect for many reasons: the acidic pH of the gastric juice induces a fast and irreversible degradation of the chlorophylls to their corresponding pheophytins and the theoretical amount of chlorophyll-bound magnesium presents in chlorophyll a is 2.72% and chlorophyll b is 2.68% of total mass. In leafy green vegetables, such as lettuce and spinach, chlorophyll-bound magnesium represents 2.5% to 10.5% of total magnesium, whereas other common green vegetables, pulses, and fruits contain <1% chlorophyll-bound magnesium. Bohn and colleagues in the conclusion section of a paper stated that “chlorophyll bound magnesium contributes a small and nutritionally insignificant part of total magnesium intake in industrialized countries” [167].

As previously mentioned, some methods of food processing, such as boiling vegetables and refining grains with the consequent removal of germ and bran, cause a substantially lower magnesium content. The loss of magnesium during food refining is considerable: white flour (−82%), polished rice (−83%), starch (−97%), and white sugar (−99%). Since 1968, a 20% decrease of magnesium content in wheat has occurred, probably due to acidic soil, yield dilution, and unbalanced crop fertilization (high levels of nitrogen, phosphorus, and potassium) [168]. The hydrosphere (i.e., sea and oceans) is the most abundant source of biologically available magnesium (about 55 mmol/L). Unrefined sea salt is indeed rich in magnesium, which represents approximately 12% of sodium mass, although refined salt, usually present in food and added for cooking either at industrial or domestic levels, lacks this mineral [6,18]. Therefore, the Western diet, characterized by easy-to-cook meals and fast food such as refined and processed food, junk food, and the near absence of legumes and seeds, predisposes apparently healthy people to magnesium deficiency.

It is important to point out that the quantification of the nutrient content in foods must be critically analyzed because nutrient bioavailability and the amount of nutrients in food portions should also be taken into consideration. Intrinsic and extrinsic factors can indeed notably affect the bioavailability of nutrients present in food- and non-food sources of nutrients [169]. Moreover, the real potential intake of the nutrient by the assumption of a determined food in a healthy and balanced diet needs indeed to be considered.

Consequently, these and other considerations must be taken into account during the consultation of nutritional tables. Selected nutritional sources of magnesium are listed in Table 3. According to data from 13 dietary surveys in nine European Union (EU) countries before Brexit, dietary intake of magnesium was estimated by EFSA, considering food consumption data from the EFSA Comprehensive European Food Consumption Database and composition data from the EFSA Food Composition Database (https://www.efsa.europa.eu/en/microstrategy/food-composition-data, accessed on February 2021)). CREA provides a list arranged by decreasing nutrient content (https://www.crea.gov.it/ accessed on February 2021), similarly to EFSA. The U.S. Department of Agriculture’s (USDA’s) Food Data Central (https://fdc.nal.usda.gov/ accessed on February 2021) lists the nutrient content of many foods and presents a comprehensive list of foods containing magnesium according to measure/portion.

Pseudo cereal and whole-grain wheat, oat, and millet were shown to be great sources of magnesium even if the cooking methods influence the real magnesium assumption per portion. For example, 100 g of wholemeal pasta cooked in water contain 42 mg of magnesium. The introduction of unrefined whole grains, nuts, legumes, and unrefined dark chocolate in the daily diet is useful to reaching a satisfactory amount of magnesium, because they represent good dietary sources of magnesium [18]. Among fruit, a high content of magnesium is found in dried apricot and dried bananas even if the normal serving of dried fruit (30 g) contains a similar amount of magnesium to a serving (100–150 g) of some fresh fruit (e.g., avocado, blackberries, prickly pears, chokecherries) [170,171].

According to the U.S. Department of Agriculture’s National Nutrient Database, Magnesium content in cocoa is at significant levels (2–4 mg/g dry powder). Therefore, a 40 g portion of 70–80%-cocoa dark chocolate would contain ≈40 mg of magnesium, enough to satisfy about ∼10% of the recommended daily allowance (300–400 mg magnesium/day in adults) [172].

It is used to say that approximately 300 mg are ingested daily in the diet, however there are several factors that hinder or facilitate magnesium availability. Unfortunately, the bioavailability studies present in the literature cover a wide range of Mg^2+^ loading administration (i.e., from <100 to >1000 mg/day) and observed different periods of time. Moreover, other important variables, such as the age of subjects (infants—adults), their physical condition, and the proximity of magnesium administration to meals and different meal matrices, did not allow for a comparison of results, leading to confusing and apparently conflicting results. Obviously, systematic studies comparing Mg^2+^ absorption efficiency between magnesium-depleted and -saturated subjects were not possible due to ethical reasons.

**Figure 2:**
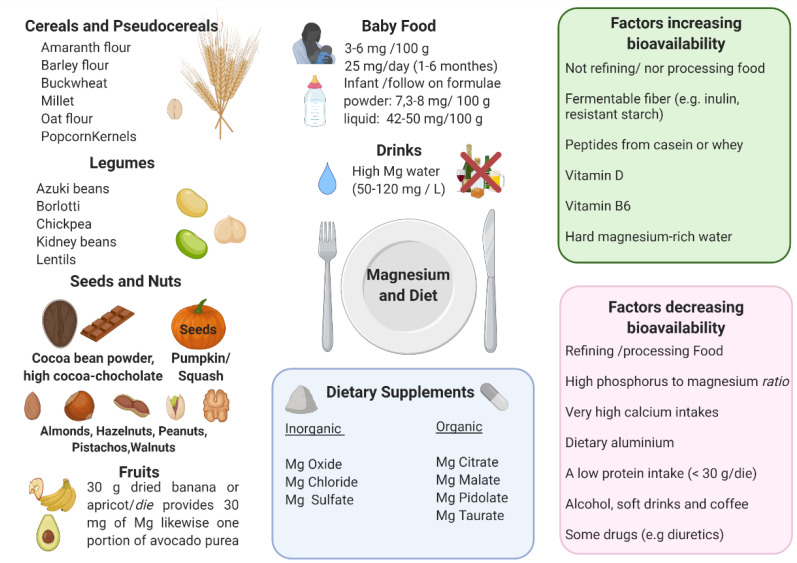
**Magnesium and Diet.** Main sources of magnesium, magnesium supplements, and factors that increase or decrease magnesium bioavailability are schematized. This image is created with BioRender.com.

Approximately 30% to 40% of dietary magnesium consumption is usually absorbed by the body. However, variables that can facilitate or obstruct magnesium absorption are here discussed and schematized in Figure 2.

In general, foods containing dietary non-fermentable fiber have indeed a high content of magnesium, nevertheless the bioavailability is low, analogously to iron [173]. By contrast, fermentable low- or indigestible carbohydrates (e.g., inulin, oligosaccharides, resistant starch, mannitol, and lactulose) enhance Mg^2+^ uptake [4].

Among the compounds that can influence magnesium absorption, there are:

− Phytates and oxalates present in foods rich in fiber can decrease the absorption of magnesium because of metal chelation. Nevertheless, the decrease of magnesium absorption caused by phytate and cellulose is usually compensated by an increased magnesium intake due to high magnesium concentrations in phytate- and cellulose-rich products [4,174,175,176,177];
− Phosphorus: high luminal concentrations of phosphates can reduce magnesium absorption, mainly because of salt formation [178]. A major source of phosphorus is represented by soft drinks: the consumption of these beverages, typically rich in phosphoric acid, has been significantly rising in the last quarter of a century. The increase in dietary phosphate is also linked to phosphate additives, present in many food items but mainly processed meats [18]. Dairy and in particular cheese have a very high phosphorus/magnesium ratio. For example, cheddar cheese has a phosphorus/magnesium ratio of ~18 and a calcium/magnesium ratio of ~26.66. On the contrary, pumpkin seeds have a phosphorus/magnesium ratio of 0.35 and a calcium/magnesium ratio of 0.21 [18];
− Very high calcium intakes can reduce the absorption of magnesium, in particular, magnesium bioavailability decreases when calcium intake is over 10 mg/kg/day [18]. Increasing evidence suggests that the optimal serum magnesium/calcium ratio is 0.4 and if it is in the range 0.36–0.28, it is considered too low. This ratio is a more practical and sensitive of magnesium status and/or turnover, than the serum magnesium level alone [12];
− Dietary aluminum may contribute to a magnesium deficit by means of an approximately 5-fold reduction of its absorption, of 41% of its retention, and by causing a reduction of magnesium in the bone. Since aluminum is widespread in modern day society (such as in cookware, deodorants, over the counter and prescription drugs, powder, baked products, and others), this could represent an important contributor to magnesium deficiency [18];
− Peptides from casein or whey could bind magnesium, which may promote absorption, analogously to other divalent cations [179]. A low protein intake (<30 g/die) could negatively influence the absorption of magnesium, however, other studies showed that magnesium use was not affected by the level of protein intake [180];
− Vitamin D seems to have a favorable role on Mg^2+^ absorption [14,85,181,182] and Mg^2+^ is important for vitamin D activation and inactivation [85];
− Vitamin B6 collaborates with magnesium in many enzyme systems and increases the accumulation of intracellular magnesium; a vitamin B6-deficient diet can lead to a negative magnesium balance via increased magnesium excretion [183,184];
− High doses of zinc can interfere with magnesium. Nielsen et al. reported that an intake of 53 mg zinc/day (4-fold higher than LARN) over 90 days can decrease magnesium balance [185];
− As for beverages, magnesium levels are decreased by excess ethanol, soft drinks, and coffee intake [186];
− Some drugs negatively affect the state of magnesium, in particular diuretics, insulin, and digitalis [23].

In turn, the magnesium content can influence the bioavailability of other nutrients: Magnesium deficiency can cause hypocalcemia [124]; Durlach suggested that the optimal dietary calcium:magnesium ratio is close to 2:1. Moreover, as previously reported, magnesium deficiency can alter the responses to vitamin D; individuals with hypocalcemia and magnesium deficiency become resistant to pharmacological doses of 1,25-dihydroxyvitamin D (active form of vitamin D), as detailed in paragraph 2.1 and recently reviewed in depth in order to highlight the role of magnesium in counteracting Covid-19 infection [85,97,187].

The magnesium content of tap/bottled water can be a significant contributor to the intake of this mineral. Tap, mineral, and bottled waters can indeed be sources of magnesium, but the amount of magnesium in water varies by source and brand (ranging from 1 mg/L to more than 120 mg/L) [188,189,190]. The magnesium in drinking water could indeed be an interesting option in order to meet the organism’s magnesium necessities, as it is highly bioavailable [191]. A study by Sabatier and colleagues demonstrated a higher magnesium bioavailability when Mg^2+^ rich mineral water was consumed during a meal [192].

Although magnesium-rich water could provide up to 30% of daily RDA, magnesium may be almost absent in soft water: the magnesium content of water tends to be ignored at the moment of purchase. Indeed, when the recommendations on the type of water for human consumption are reviewed, it is customary to downplay the importance of the magnesium it contains. The European regulations on public drinking water refer to the content of magnesium. However, as for natural mineral water, the Codex standard does not refer to magnesium content. European regulations on bottled drinking water indicate that the label can mention that it is rich in magnesium if it contains more than 50 mg/L of this mineral [*Directive 2009/54/EC of the European Parliament and of the Council of 18 June 2009 on the exploitation and marketing of natural mineral waters*].

As for the presence of magnesium in breast milk, concentrations vary over a wide range (15–64 mg/L) with a median value of 31 mg/L and 75% of reported mean concentrations below 35 mg/L [193,194].

Breastfed infants (6–24 months old) need complementary foods to satisfy 50% of their requirements for micronutrients, including magnesium, which is among those micronutrients particularly important during the sensitive period of rapid growth and development from weaning to 2 years old, and every effort should be made to ensure their adequacy in the diet [121,195].

As for baby food, in the EFSA table, it is reported that infant formulae powder contains 42 mg/100 g and 7.3 mg/100 g if considered a liquid and a slightly higher concentration is found for the follow-on formula: 8 and 50 mg/100 g for liquid and powder, respectively. In a recent study about the comparison between infants fed commercially prepared baby food and non-consumers of prepared baby food, a higher magnesium intake in the first group was reported [195].

In developed countries, children are frequently overfed and undernourished, which means “Even though children may consume an excess of energy, they may not be meeting all of their micronutrient needs” [18]. For example, more than a quarter of obese and non-obese youth do not satisfy adequate intakes of magnesium (27% and 29%, respectively), as reported by Gillis [196].

Magnesium is often added to some breakfast cereals and other fortified foods [181,197], in order to counteract deficiencies. Foods providing ≥20% of 420 mg of magnesium are considered to be high sources of this fundamental micronutrients for adults and children aged 4 years and older, as reported by the U.S. Food and Drug Administration (FDA) [198,199]. Foods providing lower percentages also contribute to a healthy diet. 

### 3.3. Nutritional and Health Claims for Magnesium

The claim **“a source of vitamins and/or minerals**” and any other claim with the same meaning for the consumer, may only be attributed to a product that contains at least a significant amount of the micronutrients, as defined in the Annex to Directive 90/496/EEC or according to Article 7 of Regulation (EC) No. 1925/2006 of the European Parliament and of the Council of 20 December 2006 on “the addition of vitamins and minerals and of certain other substances to foods”; this value for magnesium is 300 mg. Therefore, a claim that a food **is high in** magnesium and any claim likely to have the same meaning for the consumer, is authorized only if the product contains **at least twice the value** of ‘source of magnesium’ i.e., a Magnesium content ≥112 mg supplied by 100 g or 100 mL or per a single portion package, according to the Annex to Directive 90/496/EEC that report “As a rule, 15% of the recommended allowance specified in this Annex (375 mg for magnesium) is supplied by 100 g or 100 mL or per package if the package contains only a single portion should be taken into consideration in deciding what constitutes a significant amount”.

A claim stating that **the content** in one or more nutrients, other than vitamins and minerals, **has been increased,** and any claim endowed with the same meaning for the consumer, is only allowed if the product meets the conditions for the claim ‘source of’ and **the increase in content is at least 30% compared to a similar product.**

According to European Register on Nutrition and Health Claims [116], the following claims about magnesium have been authorized: Art, 13(1). The claim may be used only for food which is at least a source of magnesium as referred to in the claim “a source of” magnesium as listed in the Annex to Regulation (EC) No 1924/2006.

− “Magnesium contributes to a reduction of tiredness and fatigue”;
− “Magnesium contributes to electrolyte balance”;
− “Magnesium contributes to normal energy-yielding metabolism”;
− “Magnesium contributes to normal functioning of the nervous system”;
− “Magnesium contributes to normal muscle function”;
− “Magnesium contributes to normal protein synthesis”;
− “Magnesium contributes to normal psychological function”;
− “Magnesium contributes to the maintenance of normal bones”;
− “Magnesium contributes to the maintenance of normal teeth”;
− “Magnesium has a role in the process of cell division”.

### 3.4. Dietary Supplements of Magnesium

Magnesium supplements are available in a variety of formulations, including inorganic salt (e.g., magnesium oxide, chloride, sulfate) and organic compounds (e.g., citrate, malate, pidolate, taurate). Magnesium absorption from different kinds of supplements is not the same, nevertheless, the results obtained in the available studies in humans are hardly comparable due to the differences among the study designs. The Mg^2+^ load administered varied widely among studies (from <100 to >1000 mg/d), despite the age of subjects (infants to adults), their physical condition, or the proximity of meals to administration. In addition, the absorption depends on the magnesium status of the subjects therefore, bioavailability studies are hampered by the fact that test persons do not automatically have the same Mg status when starting the study. Moreover, due to the absence of simple, rapid, and accurate laboratory tests to measure total body magnesium stores or to evaluate its distribution in different compartments, the assessment of magnesium status after its administration is not easy to achieve. The assessment of magnesium blood level provides information about acute changes in magnesium, but blood levels are not a good marker for magnesium status and do not correlate with levels in tissue pools [200]. As a result, the data often appear confusing and conflicting. However, the amount of Mg^2+^ uptake is resulted to be dependent on the ingested dose [201]. For example, when dietary Mg^2+^ intake is low, the relative absorption rate can reach 80%, whereas it is reduced to 20% in Mg^2+^ abundance state [33]. In general, Mg^2+^ is absorbed as an ion [202,203]. Soluble forms of magnesium are more absorbed in the gut than less soluble forms [204,205]. In a recent review, Schuchardt and Hahn reported that the relative Mg^2+^ bioavailability is higher if the mineral is ingested in multiple low doses during the day rather than a single assumption of a high amount of Mg^2+^ [4]. Small studies showed that magnesium in the aspartate, chloride, citrate, and lactate salt is absorbed almost completely and is more bioavailable than magnesium oxide and magnesium sulfate [204,206]. In general, it has been suggested that absorption of organic magnesium salts is better than the absorption of inorganic compounds, whereas other studies did not find differences between salt formulations [4,200,207,208]. Unabsorbed magnesium salts in general cause diarrhea and laxative effects due to the osmotic activity in the intestine and colon and the stimulation of gastric motility [58].

Although magnesium oxide can indeed be accompanied by diarrhea with the potential to reduce magnesium absorption, it is present in mineral supplements as reviewed by Hillier [209].

Intravenous magnesium sulfate has been used as a tocolytic agent and to reduce pre-term eclampsia [210], nevertheless since magnesium chloride and sulfate have both similar and proper effects, choosing magnesium chloride seems preferable because of its more interesting clinical and pharmacological effects and its lower tissue toxicity as compared to magnesium sulfate, as reported in the review by Durlach et al. [211].

A daily supplement of 200 mg of chelated magnesium (citrate, lactate) is suggested to be likely safe, adequate, and sufficient to significantly increase serum magnesium concentration in a fasting non-hemolyzed serum sample to levels >0.85 mmol/L but <1.1 mmol/L. A steady state is usually achieved in 20–40 weeks of supplementation and is dependent on the dose [6].

Supplements may contain mixed salt of magnesium citrate and malate, with a magnesium content of 12–15%, a mixed salt formulation suggested to be used in food supplements that are intended to provide up to 300–540 mg/day magnesium. The EFSA panel concluded that it is a source from which magnesium is bioavailable, but the extent of its bioavailability still remains unclear [212].

Ates and colleagues [200] recently studied in a mouse model the distribution of different organic magnesium compounds to tissues, evaluating also the effects of different administration doses. Moreover, they evaluated the potential differences between the organic acid–bounded compounds (magnesium citrate and magnesium malate) and the amino acid–bounded compounds (magnesium acetyl taurate and magnesium glycinate), in terms of bioavailability. Magnesium acetyl taurate increases brain magnesium levels independently of dose. This observation may be due to the presence of a taurine transport system in capillary endothelial cells of the blood–brain barrier [213] that allow a rapid absorption rate even in small doses of magnesium taurate. The saturated capacity of the taurine transport system acts as a rate limiting boundary [214]. On the other hand, muscle magnesium levels increased only after a high-dose administration (405 mg/70 kg).

Another salt frequently present in supplements is magnesium pidolate. Its dissociation constant is similar to that one of the inorganic salts, therefore it is highly dissociated at physiological pH [215]. Farruggia et al. suggested that magnesium pidolate is unable to maintain the normal intracellular magnesium content in a osteoblast model when used at the concentration of 1 mM, which corresponds to the normal magnesium level in the serum [216].

The beneficial effects of magnesium supplementation appeared to be more pronounced in the elderly and alcoholics, but were not particularly apparent in athletes and physically active individuals [217]. Further research on long-term administration of different magnesium compounds and their effect on other tissues are needed.

In a placebo controlled randomized trial in adolescent girls, showing that magnesium oxide daily supplementation significantly increased BMD (bone mineral density) in one year [218]. A strong association between severe hypomagnesemia and increased risk of fractures was reported in a recent prospective cohort study that takes into consideration 2245 men over a 25-year period [219].

As deeply discussed in Section 2 and Section 5, the deficiency of magnesium is not related to a healthy status, however an indiscriminate over-treatment leading to significant hypermagnesemia must be avoided, in order to avert the risk of diseases related to the toxic effects of this mineral. Diseases associated with magnesium deficiency and toxicity are summarized in Table 4. 

If the intake slightly exceeds the daily requirement, absorption of magnesium from the gut is reduced and its active renal secretion in the urine can exceed 100% of the filtered load [6]. Although an excess of magnesium from food does not represent a health risk in healthy individuals because the kidneys eliminate excess amounts in the urine [220], high doses of magnesium from dietary supplements, drugs, or other sources can often cause not only diarrhea accompanied by nausea and abdominal cramping but also the onset of diseases [221]. As previously reported, magnesium formulations most commonly associated with diarrhea, include magnesium carbonate, chloride, gluconate, and oxide [222].

Toxic hypermagnesemia is only observed at oral magnesium doses higher than 2500 mg, i.e., doses exceeding more than 10 times the UL.

## 4. Methods to Evaluate Magnesium Status 

Since the importance of magnesium in human (and animal) health has been understood (the first review reporting the interplay between magnesium and health was published in 1965) [223], two questions have been posed: which is the correct sample reflecting the magnesium status and which is the important fraction of this element? In other words, is it better to consider the ionized free Mg^2+^ or its total amount composed by both the free ion and the fraction bound to cellular and extracellular elements [224,225]? The two questions are strictly interconnected and it is probably impossible to give a single answer because it depends on several aspects, as clearly stated by the literature [226,227,228] and resumed in the previous sections of this review.

Obviously, the easiest samples to obtain are those derived from urine or blood. Urine is relatively simple to collect, but its magnesium content is heavily affected by several factors, such as hormones or drugs, and by the complex homeostasis between the dietary intake and the mobilization mainly from bones and/or muscle [9,203,220,226,227]. Age and gender also affect urinary excretion [229,230,231,232]. Furthermore, the more reliable urine sample seems to be the 24 h collected samples [227,229,231,232], but often the urine sample consists of the first urine in the morning [233,234]. For all these reasons, urine magnesium level seems poorly correlated with the magnesium status of the body, even if it could be an important component of the “metallomic signature” in severe pathologies, such as pancreatic cancer [90].

Blood samples could consist of serum, plasma, or the corpusculated part, i.e., erythrocytes, peripheral blood mononuclear cells (PBMC), and platelets. However, several papers claimed that serum magnesium does not give an appropriated estimation of total body magnesium, being, as previously stated, around 0.3–1% of total magnesium [4,5,227]. However, the corpusculated blood constituents also represent a similarly small fraction of total magnesium, corresponding to 0.5% in the erythrocytes and an even lower fraction in PBMC or platelets. Therefore, 99% of magnesium mainly resides in bones, muscle, and soft tissues, as reported in the previous sections [4,5,227].

Tissue magnesium could represent the more reliable sample to assess, but its withdrawal could obviously be highly invasive. The analysis of fixed sublingual epithelial cells by energy dispersive x-ray microanalysis (EXA tm) is claimed to be a valid alternative to monitor magnesium status in physiological or pathological status. These cells could be easily collected by gentle scratching the sublingual tissue, then fixed on a carbon slide with cytology fixative [235,236,237].

Magnesium in the cytosol, is mainly bound to ATP, or to other phosphorylated molecules, such as phosphocreatine or inorganic phosphate. For this reason, an indirect evaluation of ionized magnesium in organs like the brain or muscle could be assessed by phosphorus magnetic resonance spectroscopy (31P-MRS). This technique enables the indirect detection of the cytosolic amount of magnesium by the shift of ATP resonance frequencies, occurring when this nucleotide is bound to Mg^2+^, and this chemical shift is a function of free Mg^2+^ concentration. This powerful technique, even if limited to the detection of this fraction, can be applied to measure free Mg^2+^ in vivo in normal [238,239,240] or pathological subjects [241,242,243,244], in several tissues, such as the brain or muscle. Recently, the application of proton NMR has been proposed for ionized magnesium evaluation in skeletal muscle [245] and, by means of clinical NMR instruments, in human plasma samples [246]. However, these techniques need very expensive and complex equipment and require very sophisticated analytical software [247], features that have confined them to very specialized laboratories.

### 4.1. Atomic Absorption Spectroscopy

To assess magnesium in biological samples, atomic absorption spectroscopy (AAS) is probably the oldest and the most widespread technique [248,249,250]. It has the important pro that could be applied to all kinds of biological sample [251,252,253], but its major cons are that sample preparation (usually acidic extracts), instrument calibration, and analysis are time expensive. Furthermore, Flame AAS raises two problems: safety as it needs dangerous gases (a mixture of air/acetylene) to burn the samples, and the sample size (millions of cells or grams of tissue). These cons are partly reduced by inductively coupled plasma-atomic emission spectrometry (ICP-AES), which allows the simultaneous multi-element analysis of small biological samples [250,253,254,255]. All these techniques are destructive and need specialized personnel to be performed. Finally, they do not allow researchers to discriminate between the free and bound form of the ion analyzed.

### 4.2. Ion Selective Electrodes 

Mg^2+^ can be measured potentiometrically using an ion selective electrode that, together with a reference electrode, forms an electrochemical system [3,256]. Ionized Mg^2+^ can be measured in whole blood [257], serum or plasma [258], or in cells as erythrocytes [251]. As in the case of Ca^2+^, several interferences, such as changes in pH, due to the loss of CO_2_, can influence the complex balance of Mg^2+^ in a serum [227,259,260]. The main disadvantages of this technique are the lack of specificity of the electrodes [261] and the rather long reaction times. However, it has significant improving potential and is spreading more and more in practice, owing to accumulating evidence regarding the presence of ionized Mg^2+^ in different clinical situations, a parameter believed, by some authors, to be more important than total magnesium content [3]. A development of this technique is represented by ion-selective microelectrodes, which can be applied to whole alive cells. In this case, the cells must be impaled on the microelectrodes, to allow for the measurement of the cytosolic concentration of Mg^2+^. A quite exhaustive discussion of this technique is presented in [256].

### 4.3. Optical Sensors

Optical chemosensors for magnesium determination represent an important and growing field of application due to their good selectivity, sensitivity, and simplicity in preparation. Several fluorimetric and colorimetric assays are proposed however, the application development seems to be more focused on fluorimetric ones than to colorimetric, maybe for the higher selectivity and sensitivity of the former [262].

#### 4.3.1. Colorimetric or Enzymatic Assay 

Colorimetric assays are commercially available and are based on the direct binding of magnesium to a chromophore, such as calmagite [262,263,264,265] or xylidyl-blue [266] or taking advantage from the enzyme which activity strictly depends on Mg^2+^. In the assay based on glycerol kinase [267], the product of the reaction (glycerol-3–phosphate) is oxidized to dihydroxyacetone phosphate and H_2_O_2_ by using glycerophosphate oxidase. Then, a peroxidase utilizes the produced H_2_O_2_ to reduce a chromogenic substrate, whose formation is proportional to magnesium concentration. Several commercial kits are available and built on patented reactives.

#### 4.3.2. Fluorescent Chemosensors

In the recent past, there has been a strong impulse in the synthesis and characterization of fluorescent dyes specific for magnesium, aiming to clarify not only the magnesium content in cells or body fluids, but also to evaluate the flows of this ion both between the intra- and extra-cellular environment and at an intracellular level [268,269,270]. Most of the commercial dyes are designed to have good specificity for the free ionized Mg^2+^, like the widely-used Mag-Fura-2, Mag-Indo-1, Mag-Fluo-3, and do not allow the detection of magnesium compartmentalization. Furthermore, they often show poor selectivity against other ions.

Several new molecules have been proposed in the past two decades, such as the family of dyes having a coumarin structure equipped with a charged β-diketone as a binding site for Mg^2+^ [271,272,273] or that based on alkoxystyryl-functionalized BODIPY fluorophores decorated with a 4-oxo-4H-quinolizine-3-carboxylic acid metal binding moiety [274]. Recently, strategies to obtain dyes capable of being addressed in specific organelles have also been proposed [275,276].

The diazacrown-hydroxyquinoline based dyes, the so-called DCHQ family, are instead specifically designed to monitor total magnesium content in cells or in cellular lysates [277,278,279]. The dye DCHQ-5, in particular, is useful in monitoring total magnesium content in whole viable cells analyzed by flow cytometric assays or in cellular lysates subjected to fluorimetric assays, starting from very small samples, up to 5 × 10^4^ cell/mL [280,281,282].

Other dyes have been synthetized and proposed and are waiting for a widespread confirmation of their biological application, as those proposed by Yadav [283].

### 4.4. Element Bioimaging 

Chemical imaging is quite a new field of study and allows for the detection of elements with high sensitivity and spatial resolution, with the further advantage that it allows for the simultaneous evaluation of several elements or molecules [283,284]. Chemical imaging techniques could provide a detailed map of elements and molecules within the cell at a nanometric/micrometric scale, opening a new window in understanding cellular functions; a detailed overview of these techniques and of the methods of samples preparations are reported in the very recent review of Decelle and colleagues [284]. In particular, metals are good subjects for x-ray fluorescence (XRF) microscopy, which exploits the excitation of the core electrons of atoms, thus generating the emission of x-rays characteristic of the elements in the sample. However, these techniques request x-ray sources, and even if laboratory sources are improving their performances in an impressive way [285], the difference in the results obtained by laboratory equipment and the synchrotron radiation sources are still noticeable [270,286].

As reported previously, the first application of x-ray detection of magnesium was proposed by Hagney and colleagues in 1995 [235]. From then, x-ray biological applications became a reality, even if they still remain a niche technique. However, it is important to report that these techniques allow researchers to simultaneously detect magnesium and other trace elements in several tissues. Clinical application of analytical scanning electron microscopy (ASEM) using computerized elemental x-ray analysis on sublingual epithelial cells was used for the simultaneous detection of magnesium and potassium in diabetes patients treated with magnesium supplementation [287]; energy dispersive x-ray spectroscopy coupled to scanning electron microscopy enables researchers to quantify differences in elemental composition in muscle biopsies of patients with peripheral artery disease (PAD), finding significative difference particularly in calcium, magnesium, and sulfur content, while C, O, K, and Na do not change within the different samples [288].

By using synchrotron sources, characterized by extremely high brilliance, a really detailed reconstruction of the map of different elements and of course of magnesium is possible, even if a routine application to clinical specimens remains obviously difficult. By this application on single cells analysis, for example, it was possible to highlight the different distribution of intracellular magnesium in cells sensitive and resistant to doxorubicin, in cells deprived of extracellular magnesium [265,289], or in cells in which different magnesium channels are modulated, as reported [265,282]. Finally, this powerful technique has recently allowed researchers to dissect the initial step of mineral deposition during osteogenic commitment, opening a window on magnesium involvement in these events [286].

## 5. Magnesium Deficiency and High Social Impact Diseases 

Over the last 30 years several, experimental, clinical, and epidemiological studies have shown that chronic magnesium deficiency is associated with and/or amplifies many major diseases [6]. Most of them are well known “social pathologies” such as diabetes, osteoporosis, and cardiovascular diseases, with a significant impact on the lives of the people affected and their families, but also on the community’s economy and social life.

The disease’s social impact can be defined as “the nexus between biological event, its perception by patient and practitioner, and the collective effort to make cognitive and policy sense out of those perceptions” [290]. Nevertheless, a comprehensive and real picture of the disease’s social impact must consider both the direct and indirect costs on the economic system. The direct costs represent the value of resources used to prevent, detect, and treat a health impairment or its effects. The indirect costs are those for employed individuals and, in the case of disabled patients, their caregivers, including the value of production lost to society due to absence from work, reduced ability, and death of productive people [291]. In fact, each pathology brings with it multiple effects, involving concentric circles of subjects, ranging from the patient directly involved, to their relational networks, to the life worlds in which it operates.

Growing scientific evidence supports the view that low intakes of magnesium could induce changes in biochemical signaling pathways, increasing the risk of illness over time. Among a few works that focus on the social impact of magnesium deficiency, a recent study is noteworthy. It asserts that subclinical magnesium deficiency increases the risk of numerous types of cardiovascular disease, burdens nations around the world with incalculable healthcare costs and suffering, and should be considered a public health crisis [18]. In this context it is important to reiterate that the acute hypomagnesemia shows clear clinical features (severe cramps, nystagmus, cardiac arrhythmias, etc.), and is easily detectable. On the contrary, subclinical or chronic magnesium deficiency is often underestimated because it reflects reduced levels of magnesium within cells and bone, not extracellular magnesium [6].

Magnesium levels should be routinely measured not only in critically ill patients but more generally in people at risk of chronic hypomagnesemia, considering that it is inexpensive to diagnose and easy to treat. This approach would permit the prevention of the onset of high social impact diseases and eventually to improve their outcome, preserving considerable resources for the whole community, such as the great savings that could be obtained by reducing the incidence and mortality due to diabetes. In fact, this illness imposes a considerable burden on society, consisting of higher medical costs, lost productivity, premature mortality, and intangible costs in the form of reduced quality of life. It has been reported that health expenses in the U.S. for diabetes increased by 26% from 2012 to 2017, passing from $245 billion to $327 billion, respectively [292]. A similar great benefit in terms of social impact could be obtained by reducing the incidence of neurological disorders, as they represent the third most common cause of disability and premature death in the EU and their burden and prevalence will increase according to the progressive ageing of the population [293].

The following section focuses on five high social impact diseases in which magnesium deficiency appears to be involved: diabetes mellitus, osteoporosis, cardiovascular diseases, cancer, and neurological disorders.

### 5.1. Diabetes Mellitus

It is well known that magnesium acts as an insulin sensitizer by inducing autophosphorylation of insulin receptors and regulating tyrosine kinase activity on these receptors [63,153,294,295]. In addition, magnesium may directly affect the activity of the glucose transporter 4 (GLUT4) and help to regulate glucose uptake into the cell [21]. Consequently, diets with higher amounts of magnesium are related to a significantly lower risk of diabetes [296]. Several studies report that a reduced intracellular magnesium level can lead to increased insulin resistance [93,297]. The incidence of hypomagnesemia in patients with type 2 diabetes is wide, ranging from 13.5–47.7% [298].

A 100 mg/day increase in total magnesium intake is reported to decrease the risk of diabetes by a statistically significant 15% [299]. Moreover, a meta-analysis of eight prospective cohort studies, involving 271,869 men and women over 4 to 18 years, showed a significant inverse association between magnesium intake from food and risk of type 2 diabetes; the relative risk reduction was 23% when the highest to lowest intakes were compared [300]. According to this, Dong et al. reported a meta-analysis of prospective cohort studies of magnesium intake and risk of type 2 diabetes included 13 studies with a total of 536,318 participants and 24,516 cases of diabetes. It was demonstrated that the magnesium intake is inversely associated with risk of contracting the disease in a dose-response manner [301]. The same conclusion was drawn from a prospective study on high risk population, involving 2582 community-dwelling participants followed up for 7 years [302]. Moreover, in a randomized controlled trial involving 116 adults with prediabetes and hypomagnesemia, the reduction of plasma glucose levels and the improvement of the glycemic status by oral magnesium supplementation were also demonstrated [303]. Additionally, a very recent trial sequential analysis confirmed that magnesium intake has an inverse dose-response association with type 2 diabetes incidence, and magnesium supplementation appears to be advisable in terms of glucose parameters in high-risk individuals [304]. 

Interestingly, some studies documented that hypomagnesemia could have an impact on many dysfunctions indicated in the pathophysiology of diabetes, such as diabetic nephropathy, poor lipid profile, and high risk of atherosclerosis, even indicating hypomagnesemia as a marker [103,305].

### 5.2. Osteoporosis

The most common bone disease in humans is osteoporosis, which represents a major public health problem and is more common in Caucasians, women, and older people [306].

It is well accepted that magnesium deficiency might represent a risk factor for osteoporosis [80,91]. Both dietary intake and supplementation of magnesium were investigated in relation to osteoporosis and risk of fractures in humans. Early works examining the effect of oral supplementation of magnesium in postmenopausal women evidenced a significant increase in BMD (bone mineral density), but the little number of enrolled subjects limited the conclusions that could be drawn [307,308]. According to one short-term study, 290 mg/day of elemental magnesium for 30 days in 20 postmenopausal women with osteoporosis counteract bone turnover and thus decreased bone loss compared with placebo [309]. Other investigations found a positive association between dietary magnesium, BMD, and lower risk of osteoporosis, suggesting that increasing magnesium intakes from food or supplements might increase BMD in postmenopausal and elderly patients [310,311].

A recent meta-analysis evidenced a positive slightly significant correlation between magnesium intake and BMD only for the femoral neck and total hip, but not for the lumbar spine [312].

Fractures and in particular osteoporotic fractures are widespread causes of disability and morbidity, especially among the aging population, and increase the burden on health systems [306]. The prevention of fractures and the evaluation of putative risk factors could be very important for the public health: serum magnesium, which may have predictive or causal relevance to the risk of fractures, could help to personalize preventive and therapeutic interventions [219]. Although several studies evidenced a positive correlation between BMD and magnesium intake, the relation to fracture outcomes is yet unclear. A prospective cohort study on 73,684 postmenopausal women showed that a lower magnesium intake is linked to decreased bone density in the hip and whole body. However this does not relate to an increase of fracture risks [313]. On the other hand, data from a large perspective study [314] and cross-sectional analysis [315] showed that by satisfying the recommended magnesium intake, the risk of fractures is lower. Accordingly, a strong association between low serum magnesium and increased risk of fractures was reported in a prospective cohort study of 2245 middle-aged Caucasian men over a 25-year period [219].

### 5.3. Cardiovascular Diseases

Increasing evidence from epidemiological studies, randomized controlled trials, and meta-analyses has shown inverse relationship between magnesium intake and cardiovascular disorders (CVD) [316]. Indeed, high magnesium intake is related to lower probability of major CV risk factors (such as hypertension and diabetes), stroke, and total CVD. In addition, a reduced risk of ischemic and coronary heart disease is related to higher levels of circulating magnesium [317]. 

It is well known that hypertension is an important risk factor for heart disease and stroke. As stated by A. Rosanoff, “Magnesium status has a direct effect upon the relaxation capability of vascular smooth muscle cells and the regulation of the cellular placement of other cations important to blood pressure—cellular sodium: potassium ratio and intracellular calcium. As a result, nutritional magnesium has both direct and indirect impacts on the regulation of blood pressure and therefore on the occurrence of hypertension” [318]. Early studies have shown that a magnesium deficiency could impact blood pressure, leading to hypertension. Oral magnesium supplementation may exert a moderate antihypertensive effect [319]. Afterwards, a meta-analysis of 12 clinical trials found that magnesium supplementation for 8–26 weeks in 545 hypertensive subjects obtained only a slight reduction in diastolic blood pressure with magnesium supplementation, ranging from nearly 243 to 973 mg/day [320]. Next, Kass et al. analyzed 22 studies with 1173 normotensive and hypertensive adults concluding that magnesium supplements for 3–24 weeks reduced both systolic and diastolic blood pressure, albeit to a small extent [321]. Other authors have pooled six prospective cohort studies including 20,119 cases and 180,566 participants. They found a statistically significant inverse association between dietary magnesium and hypertension risk without apparent evidence of heterogeneity between studies. The range of dietary magnesium intake among the included studies was 96–425 mg/day, and the follow-up ranged from 4 to 15 years [322]. Additionally, a meta-analysis on 11 randomized controlled trials counting 543 participants with preclinical or non-communicable diseases who were monitored for a range of 1–6 months, showed that the group supplemented with oral magnesium had a considerably greater decrease in blood pressure. An average reduction of 4.18 mmHg in systolic blood pressure and 2.27 mmHg in diastolic blood pressure was found after magnesium supplementation [323].

Magnesium deficiency reduces cardiac Na-K-ATPase, determining greater levels of sodium and calcium and lower levels of magnesium and potassium in the heart. Consequently, the vasoconstriction in the coronary arteries increases, inducing coronary artery spasms, heart attack, and cardiac arrhythmia [18]. Higher magnesium serum levels were significantly linked to a lower risk of CVD, as shown by a systematic review and meta-analysis of prospective studies, involving 313,041 individuals with 11,995 cardiovascular diseases, 7534 ischemic heart diseases, and 2686 fatal ischemic heart disease. Moreover, higher dietary magnesium intakes (up to approximately 250 mg/day) were correlated with a substantially lower risk of ischemic heart disease caused by a lowered blood supply to the heart muscle. Circulating serum magnesium (per 0.2 mmol/L increment) was associated with a 30% lower risk of CVD and trends toward lesser risks of ischemic heart disease and fatal ischemic heart disease [324]. In a monocentric, controlled, double-blind study, 79 patients with severe chronic heart failure under optimal medical cardiovascular treatment were randomized to receive either magnesium orotate or placebo. The two groups were similar in demographic data, duration of heart failure, and pre- and concomitant treatment. The survival rate was 75.7% compared to 51.6% under placebo, after 1 year of treatment. Clinical symptoms improved in 38.5% of patients under magnesium orotate, whereas they worsened in 56.3% of patients under placebo [325]. 

Additionally, magnesium has a well-established role in the management of torsade de pointes, a repetitive polymorphous ventricular tachycardia with prolongation of QT interval of the electrocardiogram. The guideline of the American Heart Association and the American College of Cardiology recommends intravenous administration of magnesium and potassium for the prevention and treatment of torsade de pointes, and tachycardia [326,327].

Low magnesium levels can also enhance endothelial cell dysfunction, potentially increasing the risk of atherosclerosis and thrombosis, stimulating a proatherogenic phenotype in endothelial cells [328]. The Atherosclerosis Risk in Communities study evaluated heart disease risk factors and concentrations of serum magnesium in a cohort of 14,232 white and African American men and women aged 45 to 64 years at baseline. Over an average of 12 years of follow-up, individuals with a normal physiologic range of serum magnesium (at least 0.88 mmol/L) had a 38% reduced risk of sudden cardiac death in comparison with individuals with 0.75 mmol/L or less. Nevertheless, dietary magnesium intakes did not show any risk of sudden cardiac death [329].

In an updated meta-analysis involving more than 400,000 adults from different cohorts, who were followed for 5 to 28 years, the summary estimate comparing individuals at the higher versus the lowest categories of dietary magnesium intake demonstrated a protection of 14% against the risk of CVD death. Additional assessment of the subtypes of CVD death indicated that dietary magnesium intake was inversely and significantly associated with a lower risk for heart failure and sudden cardiac death. Further dose–response analysis showed a protection of 25% in women for the increment of 100 mg/day of magnesium intake [322]. Another prospective population study of 7664 adults aged 20 to 75 years without cardiovascular disease verified the protective action of magnesium in this context: it was found that low urinary magnesium excretion levels (an indicator for low dietary magnesium intake) were related to a superior risk of ischemic heart disease over a median follow-up period of 10.5 years [330].

### 5.4. Cancer

Hypomagnesemia is also a common medical problem that contributes to the morbidity of cancer patients. Cancer is the leading cause of death worldwide; over 1.7 million people were diagnosed with cancer and over 600,000 deaths have resulted from this disease in 2018 alone [331,332].

The effects of diet in cancer metabolism are certainly an area of popular interest. A recent review highlights the mechanisms underlying magnesium disturbances due to cancer and/or its treatment [333]. Hypomagnesemia can be due to these physio-pathological mechanisms: (i) decreased intake, (ii) transcellular shift, (iii) gastrointestinal losses, and (iv) kidney losses. Moreover, cancer patients are at risk for opportunistic infections, cardiovascular complications, and are treated with classes of medications that cause or emphasize hypomagnesemia, like platinum-based chemotherapy, anti-EGFR monoclonal antibodies, and human epidermal growth factor receptor-2 target inhibitors (HER2) [334].

Several epidemiologic studies demonstrated that a diet poor in magnesium increases the risk of developing cancer, evidencing its importance in the field of hematology and oncology. Being an enzyme cofactor involved in the DNA repair mechanisms, magnesium plays a major role in maintaining genomic stability and fidelity, modulating cell cycle progression, cell proliferation, differentiation, and apoptosis. Thus, magnesium deficiency could affects these systems, leading to DNA mutations, which may result in tumorigenesis and in both the risk and prognosis of cancers [78,335]. Moreover, a protective effect of magnesium against chemical carcinogenesis has been recently reported [27].

Some studies have focused on the effect of dietary magnesium on breast cancer prognosis, suggesting that higher dietary intake is inversely associated with mortality among breast cancer patients [336]. The effect of magnesium intake on breast cancer risk has been explored, both directly and indirectly via its effect on inflammatory markers C-reactive protein and interleukin-6 [337].

Liu et al. in a recent review evidenced that magnesium supplementation can protect the liver and reduce the morbidity and mortality associated also with liver cancer. Furthermore, the risk of cancer metastasis to the liver increases in cancer patients with magnesium deficiency [338]. According to this, an in vitro study has shown that magnesium cantharidate has an inhibitory effect on human hepatoma SMMC-7721 cell proliferation by blocking the MAPK signaling pathway [339]. Moreover, magnesium administration can increase the expression of protein phosphatase magnesium dependent 1A (PPM1a), blocking TGF-β signaling by dephosphorylating of p-Smad2/3, and thus preventing the transcription of specific genes necessary for hepatocellular cancer growth [340]. 

The association between magnesium and calcium intake and colorectal cancer (CRC) recurrence and all-cause mortality was also reported. It has been observed that 25(OH)D3 and magnesium may work synergistically in decreasing the risk of all-cause mortality in these patients [341]. Higher concentrations of 25-hydroxyvitamin D3 at diagnosis are associated with a lower mortality risk in CRC patients. This is expected given the crucial roles of magnesium in several biochemical processes involved in the synthesis and metabolism of vitamin D [85,342]. In addition, in a meta-analysis that involved 3 case-control studies of colorectal adenomas and six prospective cohort studies of carcinomas, every 100 mg (4.11 mmol)/d increase in magnesium intake was associated with a 13% lower risk of colorectal adenomas and 12% lower risk of colorectal tumors [343]. Moreover, epidemiological studies have linked a magnesium deficiency with high Ca:Mg intake ratios to a higher incidence of colon cancer and mortality [342]. It has been proposed that this kind of magnesium deficiency increases intracellular calcium levels in part by increasing TRPM7 expression and unblocking the gating effect of magnesium on intracellular calcium entry. Increased intracellular calcium levels promote reactive oxygen species (ROS) generation and magnesium deficiency likely blunts cell-associated antioxidant capacity to further promote oxidative stress. This study also sheds some insight on the epidemiological findings that link high Ca:Mg ratios with increased incidence of cancer [344,345,346] and increased mortality among colon cancer patients [347].

Observational studies evidenced that elevated magnesium content in drinking water is linked with a reduced risk of esophageal cancer and decreased mortality due to prostate and ovarian cancers. Higher dietary intake of magnesium decreases the risk beyond of above-mentioned colorectal cancer, also of pancreatic cancer and lung cancer [348,349,350,351,352,353].

Although most of the literature regards solid tumors, hypomagnesemia has also been correlated with a higher viral load of the Epstein Barr virus, a virus associated with a multitude of hematologic malignancies. Studies of patients with a rare primary immunodeficiency known as XMEN disease (x-linked immunodeficiency with magnesium defect, Epstein–Barr virus (EBV) infection, and Neoplasia disease) elucidated the role of magnesium in the immune system. These patients have a mutation in the MAGT1 gene, which codes for a magnesium transporter. The mutation leads to impaired T cell activation and an increased risk of developing hematologic malignancies. Furthermore, magnesium replacement may increase the immune system’s ability to target and destroy cancer cells through this mechanism highlighted in patients with XMEN [27]. On the other hand, a very recent study has redefined MagT1 as a non-catalytic subunit of the oligosaccharyltransferase complex that facilitates Asparagine (N)-linked glycosylation of specific substrates. The authors proposed updating XMEN to “X-linked MAGT1 deficiency with increased susceptibility to EBV-infection and N-linked glycosylation defect”. However, the precise mechanism by which MAGT1 is involved in the homeostasis of magnesium and how this affects the glycosylation defect requires further investigation [354].

Moreover, a very recent work assessed the disturbance of electrolyte in leukemia. In particular, a significantly higher concentration of calcium and a lower content of magnesium in the serum and whole blood of Acute leukemia children were found, as compared to healthy subjects. Furthermore, magnesium is replaced by calcium and harmful metals (As, Cd, and Pb) which results in its deficiency, producing physiological disorders, which may be involved in acute leukemia. The level of magnesium in normal children had the range of 150–279% than AL patients [355]. This finding is consistent with other previously reported data, which indicates an association between insufficiency of magnesium and development of malignant disorders [224,356,357,358,359]. These studies highlight that a diet enriched with magnesium can decrease the incidence of cancers and the possibility that hypomagnesemia is associated with poor outcomes in cancer patients undergoing treatment.

### 5.5. Neurological Diseases

Neurological diseases are a substantial and wide spreading health burden worldwide, as shown in the Global Burden of Diseases (GBD) Study 2016. They represent the third most common cause of disability and premature death in the EU and their prevalence will presumably increase with the progressive ageing of the European population [293].

Numerous studies report the involvement of magnesium in these pathologies, the recurrent deficiency in the patients and the effectiveness of dietary integration [103,360,361]. The mechanisms by which magnesium can modulate these disorders are multiple and not fully understood. However, variation in the excitability of the central nervous system, spontaneous neuronal depolarization, and abnormal mitochondria functioning have been connected to most of them. Since glutamate is the most abundant excitatory neurotransmitter, it is often linked to etiology, prevention, and treatment of neuropathology [362,363]. For this reason, magnesium has been a potential strategy for neurological diseases mainly due to its negative modulation of the glutamatergic N-methyl-D-aspartate (NMDA) receptor. Furthermore, magnesium is a key metabolic factor in mitochondrial functioning, lowering membrane permeability and consequently reducing the possibility of spontaneous neuronal depression due to hyperexcitability [50].

A very exhaustive review describes “The Role of Magnesium in Neurological Disorders”, summarizing the recent literature on the role played by magnesium in counteracting the onset and co-treating the most frequent neurological diseases: chronic pain, migraine, stroke, epilepsy, Alzheimer’s, and Parkinson’s, as well as the commonly comorbid conditions of anxiety and depression. The authors claim that “despite to a great number of publications in this field the amount of quality data on the association of magnesium with various neurological disorders differs greatly.” Nevertheless, compelling evidence is reported about the role of magnesium in migraine and depression and for counteracting chronic pain conditions and in anxiety as well [50].

From the social impact point of view, it is worth noting that a migraine is a debilitating brain disorder with serious social and financial consequences for the individual and society. The economic impact of headache disorders is enormous in EU countries, with an annual cost of 111 billion Euros. A total of 93% of the costs are indirect and attributable to reduced productivity rather than absenteeism [364]. The serum level of magnesium in migraine patients is frequently lower than healthy subjects. Oral magnesium supplementation is prescribed for prophylaxis while intravenous magnesium administration is routinely suggested for acute migraine. The American Academy of Neurology has revealed the effectiveness of oral magnesium usage in migraine prevention [365]. The efficacy of magnesium in acute migraine treatment was confirmed by different studies [366,367,368].

Depression is a frequent and debilitating disorder that affects almost 11% of adults older than 60 and 18.8% of those younger than 60. Depression is linked to inadequate quality of life with severe impairments and is often associated with other comorbid disorders, such as anxiety and chronic pain. Interestingly, magnesium plays a role in many pathways involved in the pathophysiology of depression and it is important for the activity of several enzymes, hormones, and neurotransmitters [157,369]. Low magnesium status has been associated with increased depressive symptoms in several different age groups and ethnic populations [370,371]. Recently, it has been reported that there is a significant association between very low magnesium intake and depression, especially in younger adults [372]. Magnesium supplementation has been associated with the improvements of symptoms linked to major depression, premenstrual condition, postpartum depression, and chronic fatigue syndrome [373,374]. A recent open-label randomized trial with 126 adults comparing 248 mg of magnesium to a placebo over six weeks, showed a significant improvement of depression scores within the magnesium group within the first two weeks of treatment [375,376].

Epilepsy is a disease that affects 50 million people worldwide, characterized by seizures occurrence. Seizure activity has been strongly linked to excessive glutamatergic neurotransmission thus, magnesium could also modulate the excitotoxicity connected to epilepsy [377]. In fact, it is well known that severe hypomagnesaemia, itself, can cause seizure activity [378]. Interestingly, it has been reported that pre-eclampsia and eclampsia, conditions associated with symptomatic seizures, improved after magnesium supplementation [50].

Stroke is a cerebrovascular disease characterized by symptoms such as slurred speech, paralysis/numbness, and difficulty walking. A recent publication on stroke reviewed multiple meta-analyses and reported a dose-dependent protective effect of magnesium against stroke. Most of the meta-analyses reviewed found that each 100 mg/day increment of dietary magnesium intake provided between 2% and 13% protection against total stroke. Another updated meta-analysis, including 40 prospective cohort studies, found a 22% protection against the risk of stroke when comparing people with the highest to the lowest categories of dietary magnesium intake [322].

Alzheimer’s and Parkinson’s diseases (AD and PD) represent two aging disease of neurodegenerative character with higher social impact. The cost burden of these pathologies in European countries rises year by year, and by 2050 it will be almost two times higher in comparison with the year of 2010, estimated to reach 357 billion Euros [293,379]. 

AD is characterized by profound synapse loss and impairments of learning and memory. Excitotoxicity, neuroinflammation, and mitochondrial dysfunction have all been implicated in Alzheimer’s disease, thus, hypomagnesaemia could further hinder neuronal activity [380]. The level of magnesium in a diet is critical to support synaptic plasticity, and the decline in hippocampal synaptic connections has been associated with impaired memory [381]. Recent findings in animal studies are encouraging and provide novel insights into the neuroprotective effects of magnesium. Magnesium treatment, in fact, at an early stage may decrease the risk of cognitive decline in AD [382]. This coincides with earlier studies proving that the increase in the concentration of magnesium in the extracellular fluid results in a permanent increase in synaptic plasticity of hippocampal neurons cultured in vitro and improves learning and memory in rats [383]. Moreover, recent research suggests that ionized magnesium, cerebral spinal fluid magnesium, hair magnesium, plasma magnesium, and red blood cell magnesium concentrations are significantly reduced in AD patients compared to healthy and medical controls [21,384]. Nevertheless, the exact role of magnesium in AD pathogenesis remains unclear.

Parkinson’s disease is a common neurodegenerative disease that occurs in the *substantia nigra* and *striatum*. The exact cause of its pathological changes is still not very clear, although genetic, aging, and oxidative stress have been suggested to be linked to it. It has been shown that the concentration of magnesium in the cortex, white matter, basal ganglia, and brainstem of the PD brain is low [385,386]. However, the association between circulating magnesium and PD is still ambiguous and controversial. Human research of magnesium concentrations in PD is severely lacking, despite growing evidence implicating magnesium in animal studies [356]. The latest published study on magnesium and PD was a multicentered hospital-based case-control study that examined the dietary intake of metals in patients who were found to be within six years of onset for PD. The study found that higher magnesium concentrations were associated with a reduced risk of PD [387].

Furthermore, the involvement of magnesium in Attention-Deficit Hyperactivity Disorder (ADHD), a serious neurodevelopmental condition characterized by inattention, hyperactivity, and impulsivity, has been reported. The estimated prevalence of ADHD is between 5% and 7% in schoolchildren worldwide. Frequently, learning disorders are associated with this disease and these impairments can influence children’s quality of life and impose substantial costs on their family, health-care services, and educational systems worldwide [388]. It is well accepted that magnesium might be useful as a therapeutic agent in the treatment of ADHD because it has been reported that the serum magnesium level in ADHD children was lower than the controls [389,390]. Moreover, magnesium supplementation (alone or in combination with vitamins or other metals) significantly improved ADHD symptoms [391,392]. Magnesium supplementation along with standard treatment ameliorated inattention, hyperactivity, impulsivity, opposition, and conceptual level in children with ADHD. A very recent paper assessed that magnesium and vitamin D supplementation in children with ADHD disorder was effective on conduct problems, social problems, and anxiety/shy scores compared with placebo intake [381,388].

## 6. Conclusions

This multifaceted analysis of the importance of magnesium for maintaining a good state of health, starting from the tuning role played by this element at cellular level, revealed the importance of disseminating dietary strategies that satisfy the recommended daily value. Moreover, it is fundamental to have reliable and minimally invasive methods either to promptly identify magnesium deficiency in various body districts or to accurately monitor the efficacy of supplements to prevent and counteract diseases that correlate with magnesium deficiency. Indeed, magnesium has to be considered as a real metabolite instead of a simple electrolyte and its deficiency has a great impact on different physiological functions. 

Data from many studies indicate that in about 60% of adults, magnesium intakes from the diet is insufficient and that subclinical magnesium deficiency is a widely diffused condition in the western population. Hence, more attention should be paid to the preventive role of magnesium for social pathologies, encouraging a more adequate dietary intake of the cation and supplementations. As extensively described above, magnesium is found in a wide variety of non-refined foods and is among the less expensive available supplements. Moreover, magnesium trials have shown that magnesium supplements are well tolerated and generally improve multiple markers of disease status.

## Figures and Tables

**Table 1:** Enzymes requiring magnesium.

LOCALIZATION	ENZYME	Mg-ATP^2−^	FREE Mg^2+^	REF
Cytosol: glycolytic pathway	Hexokinase		-	[53]
	Phosphofructokinase		-	
	Phosphoglycerate kinase		-	
	Pyruvate kinase		-	
	Aldolase	-		
	Enolase	-		
Mitochondrion	Pyruvate dehydrogenase phosphatase	-		[65]
	Isocitrate dehydrogenase	-		[55]
	α-Ketoglutarate dehydrogenase	-		[56]
	F_o_/F_1_-ATPase	-		[58]
Muscle cytosol/Heart mitochondrion	Creatine kinase		-	[53]
Liver, cytosol	Phosphoenolpyruvate carboxykinase	-		[40]
	Glucose-6-phosphatase	-		
β-subunit of the insulin receptor	Receptor tyrosine kinase activity		-	[62,63]

**Table 2:** Magnesium intake recommendations expressed in terms of: Population Reference Intake (PRI), Average Requirement (AR), Recommended Dietary Allowance (RDAs)—Dietary Reference Intakes (DRIs), Dietary Reference Values (DRVs)—Adequate Intake (AI), “Livelli di Assunzione di Riferimento di Nutrienti ed energia per la popolazione italiana” (LARN) and **tolerable** Upper intake Level (UL).

Life Stage	PRI (mg)	AR (mg)	UL * (mg)	RDA-DRI (mg)	DRV-AI (mg)	LARN (mg)
Birth to 6 months	-		Nd	30		
Infants 7–12 months	80	Nd	Nd	75	80	80
Children 1–3 years	80	65	250	80	170	80
Children 4–6 years	100	85	250	130	230	100
Children 7–10 years	150	130	250	240	230	150
Teen boys 11–18 years	240	170–200	250	410	300	240
Teen girls 11–18 years	240	170–200	250	360	250	240
Men	240	170	250	400–420	350	240
Women	240	170	250	310–320	300	240
Pregnant	240	170	250	350–400	300	240
Breastfeeding	240	170	250	310–360	300	240

* the UL value refers to the magnesium taken in pharmaceutical or supplement form, in addition to magnesium content already present in the diet.

**Table 3:** Magnesium content in Food according to the EFSA Comprehensive European Food Consumption Database (CREA) and U.S. Department of Agriculture, Agricultural Research Service—Food Data Central.

Food	EFSA (mg/100 g)	CREA (mg/100 g)	USDA (mg/Measure)	Measure and Weight
Wheat/Cereal bran	451	550	354	1 cup, 50 g
Pumpkin and squash seed, dried	429	592	764	1 cup, 46 g
Cocoa powder	545	499	29	1 ts ^1^, 6 g
Sunflower seeds dried	346	n.a ^2^	173	1 cup, 130 g
Wheat germ	276	255	275	1 cup, 115 g
Amaranth flour	266	266	476	1 cup, 193 g
Cashews dried	258	260	352	1 cup, 137 g
Sweet, dried almonds	251	264	386	1 cup, 143 g
Peanuts, roasted	229	175	260	1 cup, 146 g
Quinoa	n.a	189	335	1 cup, 170 g
Pecans	168	121	132	1 cup, 109 g
Hazelnuts, dried	163	163	187	1 cup, 187 g
Beans, dried	158	170	258	1 cup, 184 g
Walnuts, dried	150	158	185	1 cup, 169 g
Chickpeas, dried	150	131	158	1 cup, 100 g
Pistachios, dried	147	160	149	1 cup, 123 g
Millet, shelled	136	160	228	1 cup, 200 g
Wheat flour, hard	136	120	164	1 cup, 120 g
Oat flour	131	n.a ^2^	150	1 cup, 169 g
Buckwheat flour, whole-groats	121	231	301	1 cup, 120 g
Macadamia	115	120	156	1 cup, 132 g
Wholemeal pasta	111	101	95	1 cup, 90 g
Lentils, dried	101	83.1	113	1 cup, 100 g

^1^ Ts: Teaspoon; ^2^ n.a: Not available data.

**Table 4:** Diseases associated with magnesium deficiency and toxicity.

Magnesium Deficiency	Magnesium Toxicity
Hypocalcemia, hypokalemia	Diarrhea, nausea and vomiting
Osteoporosis	Muscle weakness
Cardiovascular disorders	Low blood pressure
Neurological disorders	Loss of deep tendon reflexes
Diabetes	Sinoatrial or atrioventricular node blocks
Tumors	Respiratory paralysis
Covid-19	Cardiac arrest

## Data Availability

Not applicable.
